# Recycling of waste glass as aggregate in cement-based materials

**DOI:** 10.1016/j.ese.2020.100064

**Published:** 2020-11-02

**Authors:** Edward Harrison, Aydin Berenjian, Mostafa Seifan

**Affiliations:** School of Engineering, Faculty of Science and Engineering, The University of Waikato, Hamilton, New Zealand

**Keywords:** Waste glass, Fine aggregate, Sand replacement, Pozzolanic reactivity, Cement, Recycling alternative

## Abstract

Glass is a common material made from natural resources such as sand. Although much of the waste glass is recycled to make new glass products, a large proportion is still being sent to landfill. Glass is a useful resource that is non-biodegradable, occupying valuable landfill space. To combat the waste glass that is heading to landfill, alternative recycling forms need to be investigated. The construction industry is one of the largest CO_2_ emitters in the world, producing up to 8% of the global CO_2_ to produce cement. The use of sand largely depletes natural resources for the creation of mortars or concretes. This review explores the possibilities of incorporating waste glass into cement-based materials. It was found waste glass is unsuitable as a raw material replacement to produce clinker and as a coarse aggregate, due to a liquid state being produced in the kiln and the smooth surface area, respectively. Promising results were found when incorporating fine particles of glass in cement-based materials due to the favourable pozzolanic reaction which benefits the mechanical properties. It was found that 20% of cement can be replaced with waste glass of 20 μm without detrimental effects on the mechanical properties. Replacements higher than 30% can cause negative impacts as insufficient amounts of CaCO_3_ remain to react with the silica from the glass, known as the dilution effect. As the fine aggregate replacement for waste glass increases over 20%, the mechanical properties decrease proportionally; however, up to 20% has similar results to traditionally mixes.

## Highlights

• Pozzolanic reaction exhibited by glass can improve the materials properties.
• 20 μm waste glass at 20% cement replacement obtains optimal results.
• 20% fine aggregate for waste glass obtains satisfactory results.
• Waste glass provides a poor coarse aggregate due to the smooth surface of glass.

## Abbreviation definition

AlO: Aluminium oxide
ASR: Alkili-silica reaction
C_3_A: Calcium aluminates
C_3_S: Tricalcium silicates
CaCO_3_: Calcium Carbonate
CaO: Calcium oxide
CO_2_: Carbon Dioxide
FA: Fly Ash
GBFS: Granulated Blast Furnace Slag
GP: Glass powder
NS: Natural sand
SO: Sodium oxide
SO_2_: Silica Dioxide
WG: Waste glass

## 1 Introduction

A huge problem with glass is the single-use purpose for applications such as beverage bottles, which is one of the main uses of glass. Waste glass is estimated to account for 5% of the global municipal solid waste generated in 2016 [1], with recycling rates varying globally and within regions. In 2017, Europe had a glass recycling rate of 71.48%, with individual countries varying from 98% (Slovenia and Belgium) to 9% (Turkey) [2]. The United States had a 26.63% glass recycling rate in 2017, disposing of 52.9% of glass containers generated to landfill [3]. Glass waste is non-biodegradable, permanently filling up valuable space in landfills. The disposal of glass waste to landfills due to poor recycling practices increases the dependency on natural resources, depleting sources such as beaches to produce more glass products. As there becomes an increasing demand for landfill space, landfill tax is likely to rise to urge better recycling habits. Finding alternative methods for reusing waste glass can cut disposal costs while ensuring the longevity of landfills and natural resources.

Alternative to landfill, glass can be repurposed by two systems: closed-loop or open-loop. A closed-looped system is where the glass can be recycled by either reusing the glass containers by operating a return scheme, or manufacturing new glass products from the recycled glass cullet [4]. For every 10% of glass cullet used (by weight) in the production of glass, carbon emissions and energy consumption are reduced by 5% and 3%, respectively [5]. This is the ideal form of glass recycling as glass can be recycled indefinitely if it avoids contamination, including food-waste contamination or cross-colour contamination. Re-processors set acceptable limits for contamination in the glass cullet to ensure the quality of the final product. Amber and green container glass include additives which can lower the quality of clear container glass or discolour the final product, so it is important to avoid the mixing of different coloured glass [6]. Due to this, colour sorting of glass containers proves to be an important step in the glass recycling process, sorting between clear, green, and amber glass containers.

An open-looped system is where the waste glass is repurposed into alternative products that are then disposed of at the end of use [4]. Such materials can include ceiling insulation which is commonly made from glass fibres [7]. Open-loop systems are the least ideal form of recycling as it ends the continuing recycling process. However, if the glass waste is not colour sorted or the quality is compromised, other forms of glass recycling often cannot proceed.

Another form of open-loop recycling for glass waste can be in the construction industry by the implementation in cement-based materials. Waste glass can be used as partial clinker replacement in the production of cement, finely grinded glass can be used for its pozzolanic properties by producing Portland-cement blends, or filler replacement in cement-based materials as fine or coarse aggregate in mortar or concrete. The cement industry is found to contributes to 8% of the global emissions, with 90% of that being due to the production of clinker [8,9]. Portland cement is manufactured by combining grounded clinker with gypsum, an agent used to control the setting rate of the cement. The process of producing clinker is emission heavy as it requires intense heating to facilitate calcination, which is found to account for half of the emissions in the production of cement.

Proper chemical proportions of predominantly limestone (source of CaCO_3_) and clay (a source of SiO_2,_ and Al_2_O_3_) are milled together with other materials such as shale (source of Fe_2_O_3_) to form the raw meal [10]. The raw meal is then heated in a kiln at high temperatures (900 °C) to allow for the calcination of the CaCO_3_ to form calcium oxide (CaO), while discharging a by-product of carbon dioxide (CO_2_) [11]. Temperatures are increased to 1450 °C to allow for minerals such as calcium silicates (3CaO·SiO_2_, C_3_S; and 2CaO·SiO_2_, C_2_S), tricalcium aluminate (3CaO·Al_2_O_3_, C_3_A) and tetra-calcium aluminoferrite (4CaO·Al_2_O_3_·Fe_2_O_3_, C_4_AF) to form and become partially molten to physically combine and form the clinker granules.

Incorporating waste glass in cement-based materials has the potential for creating a more sustainable future. Currently, trials of waste glass being used in cement-based materials on the commercial scale are limited; however, a few cases can be found, including the 2012 Queen Elizbeth Olympic Park. The Olympic Delivery Authority (ODA) set sustainability as a key focus, aiming to reduce the concrete carbon foot print by 25% [12]. Due to the potential loss of strength, glass sand substituted fine aggregate at replacements of 0–15% depending on the required strength designation, on average 2% of all fine aggregate used was substituted for glass. Another example is the Lion Nathan brewery which finished construction in 2010. The Lion brewery incorporated 3500 tonnes of waste glass within the concrete mix, using over 1.3 million recycled beer bottles [13]. However, the high silica content of glass saw the potential for alkali-silica reaction (ASR), which can cause swelling of the concrete leading to the formation of cracks. Preventing this required 12 months of lab research and testing to get the correct mix proportions to successfully complete the project [14]. This review will explore the past research into the uses of waste glass in cement-based materials and the different effects that have been found to investigate the viability of this recycling method.

## 2 Characteristics of glass and methods of processing crushed glass

### 2.1 Physical and chemical characteristics of glass

Waste glass can be categorized by three main types, soda-lime glass, borosilicate glass, and lead glass with their chemical compositions displayed in Table 1. Soda-lime glass is generally used to make container glass (e.g. beverage bottles) and plate glass (e.g. windowpanes), with borosilicate glass being used for insulation and laboratory glassware, and lead glass being used for domestic glassware. Soda-lime glass is the key focus as glass containers made from soda-lime glass make up the predominant source of waste glass. Additives can be incorporated into the glass mix to improve properties or alter the glass color from clear to amber, green, or other colours.

**Table 1:** Chemical composition of different glass types [15].

Chemical Compositions [%]	Soda-lime Glass			Borosilicate Glass	Lead Glass
	Clear	Amber	Green		
SiO_2_	73.2–73.5	71.9–72.4	71.3	70–80	54–65
Na_2_O + K_2_O	13.6–14.1	13.8–14.4	13.1	4–8	13–15
Al_2_O_3_	1.7–1.9	1.7–1.8	2.2	7	–
MgO + CaO	10.7–10.8	11.6	12.2	–	–
SO_3_	0.20–0.24	0.12–0.14	0.05	–	–
Fe_2_O_3_	0.04–0.05	0.30	0.56	–	–
Cr_2_O_3_	–	0.01	0.43	–	–
B_2_O_3_	–	–	–	7–15	–
PbO	–	–	–	–	25–30

**Fig. 1:**
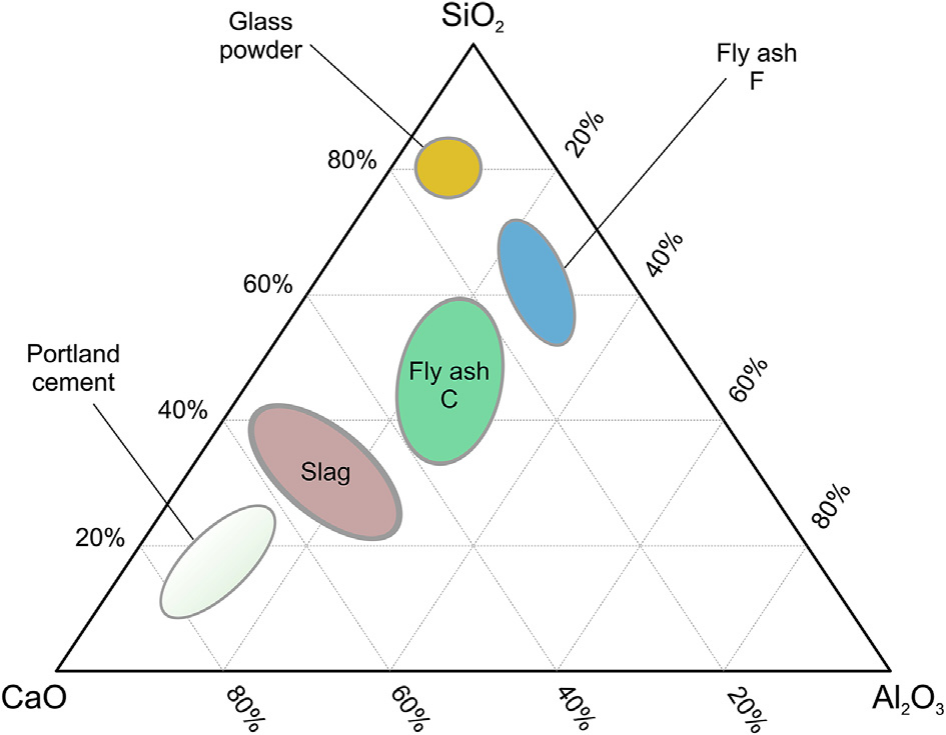
CaO-AL_2_O_3_–SiO_2_ ternary diagram of Portland cement, FA class F and C, GBFS, and glass powder.

When looking at the CaO–Al_2_O_3_–SiO_2_ ternary diagram in Fig. 1, it can be seen why glass has the potential to be incorporated into cement-based materials. SiO_2_ is the main reactive component in the pozzolanic reaction, with glass containing the most amount of by composition.

### 2.2 Processing crushed glass

Glass can be recycled and used to produce additional glass products in an indefinite closed-loop cycle. The recycling process begins by collection, where glass types and colours are separated to ensure the correct chemical composition for the intended use. The recycled glass must be free of other contaminants such as food waste, dirt, and ceramics as these may cause impurities in the final product. The crushed glass can then be mixed with raw materials then heated in the manufacturing kiln at high temperatures (1200–1400 °C) to melt the glass mix [15]. The liquid glass mix then leaves the kiln to be blown or pressed into new glass products. The process of manufacturing glass is resource and energy heavy but incorporating waste glass can cut the consumption of these down. The use of 10% crushed glass by weight in the manufacturing process can reduce the required raw materials by 5% [5].

## 3 Waste glass in cement materials

Due to the chemical composition and potential pozzolanic activity exhibited by glass, waste glass has the potential to be utilized as a partial clinker replacement or a cement additive to produce Portland cement blends. This section investigates the effects that have been found in terms of mechanical properties due to waste glass being used in these two applications.

Xie and Xi investigated the use of waste glass as a potential clay substitute in the production of clinker [16]. They found that glass would have a liquid phase in the kiln which would have adverse consequences on the operation. Although the materials are both similar in chemical composition, they found a decrease in tricalcium silicates (C_3_S) was forming, the main bonding ingredient in the clinker. It was also concluded that substituting waste glass for clay produced an undesirable reactive compound (NC_8_A_3_) forming from the alkali found in the glass (sodium oxide) reacting with the spare calcium aluminates (C_3_A). This compound causes flash setting and poor strength of the hardened cement.

However, further researchers investigated the possibility of reducing the required clinker for cement by partially replacing clinker for waste glass. Dvořák et al. found that the pozzolanic properties of fine glass to be high and can be further increased by milling or mechanical activation in high speed grinding mills [17]. They also found that co-milling the clinker and glass prevented the formation of glass agglomerates so that the glass can be used for its pozzolanic properties and rather than just the physico-mechanical properties as filler. This is further supported by Halbiniak’s and Major’s findings, concluding that grinding green or clear waste glass with clinker at 7.5% and 15% replacement (by weight) showed a higher compressive strength after 2, 7, and 28 days than the control of pure cement [18]. Separately grinding the glass and clinker showed similar results, however, a slight decrease in compressive strength from the co-grinded samples. All samples containing waste glass were found to produce higher compressive strengths than the control samples, with samples comprising of 15% clear glass co-grinded with clinker producing the highest compressive strength at 28-days (41.2 MPa compared to the control of 36.8 MPa).

These researchers have found that waste glass can be utilized to limit the requirement of clinker and be used as a partial replacement. However, it is unsuitable in the replacement of raw materials to produce clinker. The incorporation of waste glass would minimise the required clinker which would reduce the CO_2_ emissions produced and lower the total cost. A summary of the effects can be found in Table 2.

**Table 2:** Summary of the effects on waste glass to produce Portland cement blends.

Comparison	Replacement [%]	Particle Size [μm]	Compressive Strength			Ref.
			3 Days	7 Days	28 Days	
Separate vs Co-milling	20	–	↑	↑	↑	[17]
Separate vs Co-milling	7.5, 15	20	↑	↑	↑	[18]
Control vs WG			↑	↑	↑	

If not replacing pure clinker to form a cement blend, waster glass can be used for its pozzolanic properties as a partial cement replacement. Reducing the amount of required cement by partially substituting it for different pozzolanic material is another viable option to reduce the emissions in the construction industry. Currently, fly ash (FA) or granulated blast-furnace slag (GBFS) blends of Portland cement are quite popular; however, another possibility could be the use of waste glass. Introducing waste glass as a pozzolanic activity promoter will reduce the amount of required cement while lessening the need for other materials such as FA or GBFS.

**Fig. 2:**
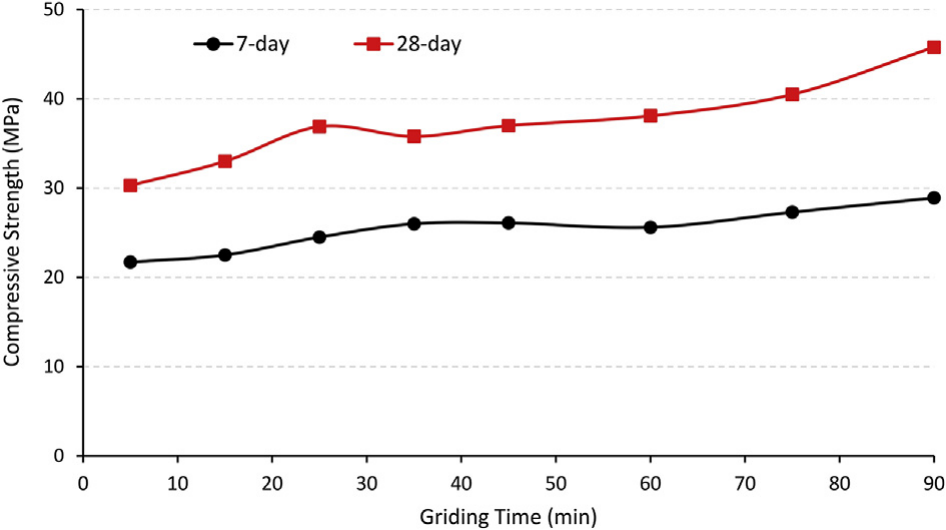
Effects of grinding duration on the compressive strength at 7 and 28 days.

Ali et al. [19] found that finer waste glass particles increases the pozzolanic activity, where samples containing waste glass of 40 μm, 20 μm, and 10 μm at the same replacement produced 28-day compressive strengths of 51.5 MPa, 55.5 MPa, and 66.6 MPa, respectively. This is supported by Shi et al. [20] who found samples containing waste glass with finer particle distributions produced higher compressive strengths at 28 days than those with coarser particle distributions. Z. Chen’s [21] research showed similar results, where increasing the grinding time of waste glass generally increased the compressive strength of the samples at 7 and 28 days (illustrated in Fig. 2).

Letelier et al. [22] found 38 μm to be the maximum size to allow for a 30% replacement of cement (by weight) without adverse effects on the mechanical properties, supported by Shao et al. [23] who found 38 μm allows for a 30% replacement by volume. This is further supported by Khmiri’s et al. findings [24], where it was found that waste glass of 40 μm decrease the compressive strength, however, up to 20% cement replacement (by weight) of waste glass grounded to 20 μm can improve the late mechanical properties. Mostofinejad et al. [25] found that 30% replacement of cement for milled waste glass by weight can significantly lower the compressive strengths at 28 and 300 days, having a strength reduction of 40% and 42%, respectively. Letelier’s and Al-Hashmi’s results show 38 μm of waste glass at 20% cement replacement (by weight) to have the optimal mechanical properties, with a 30% cement replacement having the lowest embodied CO_2_. Letelier found a replacement of 20% at 38 μm to have the greatest compressive strength increase from 28 days to 90 days, with samples of the same particle size at replacements of 10% and 30% showing similar strength increases to the reference sample. Glass powder replacement of up to 20% (by weight) cement is also found to be the optimal dosage by Z. Ismail [26] and Tejaswi et al. [27], who found although it decreased the compressive strength at 7 and 28 days, it was higher than the allowable strength index of 75%. Shayan [28] found a replacement of cement by weight for waste glass powder (92.4% < 15 μm) decreases the 28 day compressive strength, with an increasingly adverse effect as the replacement percentage increases.

Huseien et al. [29] investigated the effects of glass bottles waste nano powder (BGWNP at 80 nm) when incorporated in alkali-activated mortar blends as a GBFS partial replacement. Huseien had a FA to GBFS ratio of 70:30 as a reference sample, and tested samples of GBFS:BGWNP ratios of 25:5, 20:10, 15:15, and 10:20. At 28 days, it was found the 5% and 10% BGWNP samples increase the compressive, flexural, and tensile strengths with the 5% replacement being the optimum dosage; while the 15% and 20% BGWNP samples both decreased these strengths.

The following researchers compare the effects of glass powder to fly ash on the strength of cement mortars. Shao et al. found that waste glass of 38 μm at 30% cement replacement (by volume) produces a higher compressive strength than fly ash at the same cement replacement [23]. This is further supported by Shi et al., who found fly ash to have less pozzolanic activity than samples containing finely milled glass particles [20]. The glass sample with the highest distribution of coarse glass particles was found to have the lowest compressive strength. However, the three samples with a finer glass particle distribution displayed higher pozzolanic reactivity than FA, with the two samples with a particle distribution similar to cement powder having the potential to achieve a higher compressive strength than 100% cement with a cement replacement of up to 20% (by weight). This is contrary to Tamanna’s findings [30], who found cement replacements for waste glass at 10%, 20%, and 30% (by weight) produced lower compressive strengths than that of the control sample, while the standard FA-cement blend produced higher compressive strengths. Similar results were found for the flexural and tensile strengths, where increasing the replacement decreases the strength. It was found particle size and dosage of waste glass to be two critical variables that can have a significant impact on the mechanical properties of the hardened material. It can be determined that lower replacements of cement with fine particles of waste glass can improve the mechanical properties of the material by utilizing the pozzolanic activity from the glass. As replacements of cement are increased, the decreasing effect caused by removing cement powder can surpass the positive effects due to the pozzolanic activity, therefore decreasing the mechanical properties of the material. Coarse particles of waste glass reduce the amount of surface area for the pozzolanic reactants to form, causing the waste glass to behave predominantly as a filler material rather than as a pozzolanic. A summary of the varying results can be found in Table 3.

**Table 3:** Summary of effects of waste glass as a cement additive.

Replacement [%]	Particle Size [μm]	Compressive Strength				Flexural Strength				Tensile Strength				Ref.	Notes
		3	7	28	>28	3	7	28	>28	3	7	28	>28		
20	80% < 150	↓	↓	↓	–	–	–	–	–	–	–	–	–	[20]	
	90% < 90	↑	↑	↑											
20	20	↓	↓	↓	–	–	–	–	–	–	–	–	–	[19]	
30, 40	20	↓↓	↓↓	↓↓	–	–	–	–	–						
10	38	–	↓	↓	↓	–	↓	↓	↓	–	–	–	–	[22]	90 days
20			↓	↑	↑		↑	↑	↓						
30			↓	↑	↓		↓	↓	↓						
10	45		↓	↑	∼		↑	↑	↓						
20			↓	↓	↓		↑	↑	↓						
30			↓	↓	↓		↓	↓	↓						
10	75		↓	↑	∼		↓	↓	↓						
20, 30			↓	↓	↓		↓	↓	↓						
30	38	↓	↓	↓	↑	–	–	–	–	–	–	–	–	[23]	90 days
	75	↓	↓	↓	↓										
	150	↓	↓	↓	↓										
20	20	–	↓	↓	↑	–	–	–	–	–	–	–	–	[24]	90 days
	40		↓	↓	↓										
30	<75	–	–	↓	↓	–	–	–	–	–	–	–	–	[25]	300 days
20	68% < 1180	–	↓	↓	–	–	–	–	–	–	–	–	–	[26]	
20	150	↓	↓	∼	–	–	–	–	–	–	–	–	–	[27]	
10, 30, 40, 50		↓↓	↓↓	↓↓											
10, 20, 30, 40	92.4% < 15	–	–	↓	–	–	–	–	–	–	–	–	–	[28]	
5, 10	50% < 0.08	–	–	↑	–	–	–	↑	–	–	–	↑	–	[29]	
15, 20				↓				↓				↓			
10, 20, 30	90% < 11.6	–	↓	↓	↓	–	–	↓	–	–	–	↓	–	[30]	56 days

‘↑’ denotes sample improved relative to reference sample; ‘↓’ denotes sample decreased; ‘∼‘denotes negligible results to reference.

## 4 Waste glass as an aggregate replacement for the use in mortar or concretes

Another viable option for the utilisation of waste glass in cement-based materials is for the replacement of fine and coarse aggregate. The use of natural aggregate promotes the depletion of sources such as beaches and quarries which cannot be sustainable. Therefore, other means should be investigated, opening the potential for waste materials, including waste glass. This section will explore the past research which has gone into both these options to understand what has been found in terms of the mechanical properties and overall benefit from the substitution of traditional aggregates for waste glass.

### 4.1 Fine aggregate replacement

#### 4.1.1 Effects of fine aggregate replacement on the mechanical properties

Penacho et al. [31], who found replacements of sand for 20%, 50%, and 100% glass (by volume) with similar particle distributions to sand produced higher compressive and flexural strengths; however, a replacement of 100% produced a lower compressive strength at 28 days. Penacho found samples with higher replacements of sand for fine waste glass produce a greater increase in strength from 28 days to 90 days, with the replacement of 100% sand for waste glass surpassing the reference sample at 90 days. These increases in strength are attributed to the positive pozzolanic reaction which occurs with fine glass particles. Tamanna [32] found 20% replacement by weight of coarse sand for 3000 μm waste glass produced higher compressive strengths at 7, 28, and 56 days, while replacements of 40% and 60% had decreasing effects on the compressive strength. The samples generally showed an increase in flexural and tensile strengths at 28 days, while the 40% sample showed a slight decrease in flexural strength and the 60% sample showed a noticeable decrease in tensile strength. Tamanna found the 40% replacement series to have the greatest increase in compressive strength from 28 days to 56 days; however, the 20% and 60% series had a lower compressive strength increase than the control.

**Fig. 3:**
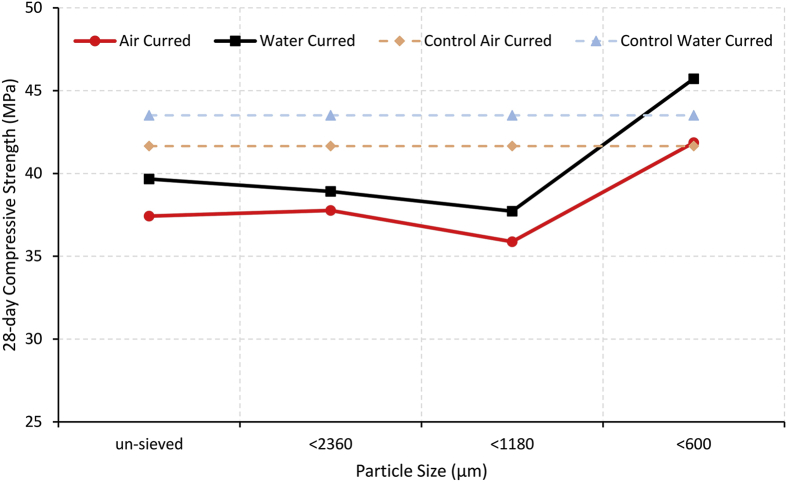
Effects of particle size and curing method on the 28-day compressive strength.

Corinaldesi et al. [33] analysed the compressive and flexural strengths at 180 days to examine the effects of particle size, testing samples consisting of glass particles less than 36 μm, 36–50 μm, and 50–100 μm. Corinaldesi found increasing particle size at a 30% fine aggregate replacement (by weight) decreases the compressive strength; however, the samples still show improvements relative to the reference sample. When the replacement is increased to 70%, the 36–50 μm samples show the highest compressive strength, followed by the 36 μm sample. The 180-day flexural strength results show negligible changes; however, the 50–100 μm samples show slight increases to the reference sample. Lee et al. [34] investigated the effects of the glass waste particle size, finding samples consisting of particles less than 600 μm improve the compressive strength at 28 days. Fig. 3 illustrates this effect being magnified by water curing, with the 600 μm samples having the largest increase in strength between curing methods.

Batayneh et al. [35] found increasing the replacement of waste glass with the incorporation of FA increased the compressive strength; however, has no effect on the splitting strength. Gerges et al. [36] found that the replacement ratio of sand for fine glass has negligible effects on the compressive, splitting tensile, or flexural strengths.

**Fig. 4:**
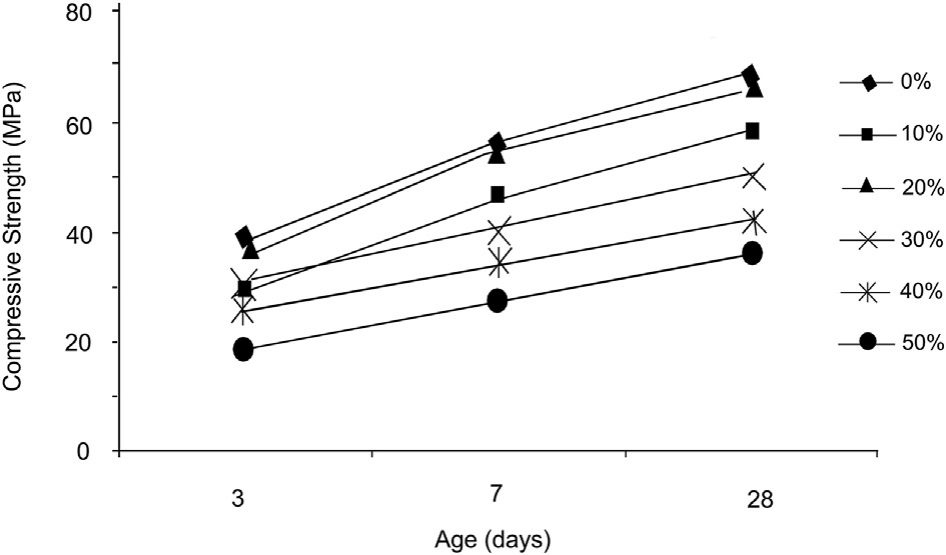
Compressive strength of concrete when sand is partially replaced with waste glass [27].

C. Chen et al. [37] researched the effects of incorporating electronic-grade glass into concrete, where it was found up to 40% replacement (by weight) of 75 μm E glass had significant improvements on the compressive strength. Tejaswi et al. [27] found replacement of 20% fine aggregate by weight can produce similar compressive strengths to the control, while replacement of 10%, 30%, 40%, and 50% produce lower compressive strengths, illustrated in Fig. 4.

**Fig. 5:**
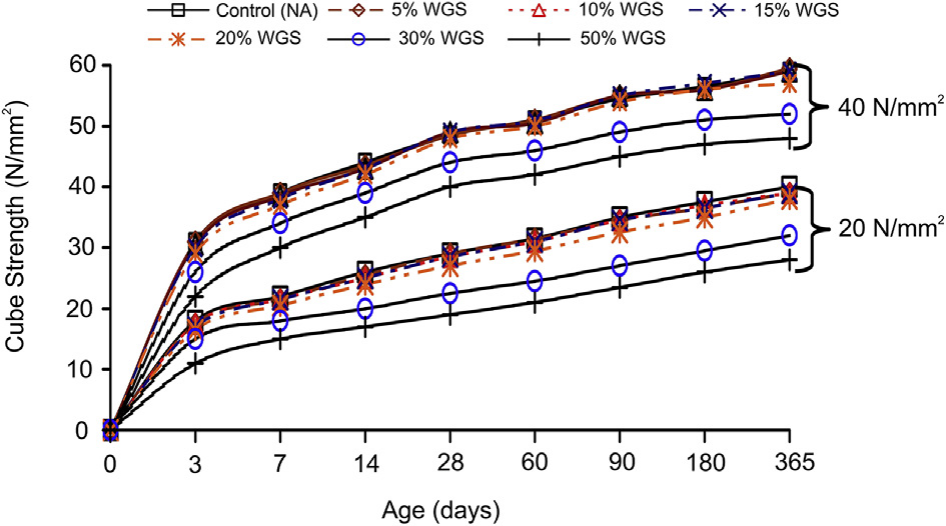
Effects of different sand replacements for waste glass on the compressive strength at various days [46].

On the contrary, Park et al. [38], Ling and Poon [39], Ali and Al-Tersawy [40], Mardani-Aghabaglou et al. [41], Sikora et al. [42], Tan and Du [43], and Tuaum et al. [44] found increasing the replacement of sand for fine waste glass has an increasingly adverse effect on the mechanical properties, particularly the compressive strength. Glass aggregate is smooth, therefore reduces the bond strength between the cement paste and recycled glass. Afshinnia’s and Rangaraju’s [45], research shows using glass powder as a 20% replacement (by weight) for aggregate reduces the 28-day compressive strength by 15% when using traditional aggregate. This can be partially attributed to the fineness of the waste powder being 17 μm, significantly decreasing the water to powder ratio. Limbachiya [46] and Ismail and Al-Hashmi [26] found up to 20% of sand can be replaced (by weight) without significantly impacting the compressive strength, while Shayan [28] reports up to 30% of aggregate can be replaced (by weight) for glass powder without significantly damaging the long-term mechanical properties. Park et al. [38] found that at 30% replacement of sand for waste glass (by weight) produces a 3% decrease in compressive strength, still being in the tolerable levels for structural applications. Sikora et al. [42] found a 25% fine aggregate replacement for waste glass (by weight) has a decrease in early compressive strength (2–7 days), however, an increase in the 28-day compressive strength. From Sikora’s et al. findings, the inverse is true for the flexural strength. Limbachiya [46] investigated the late-day strength development by also testing samples beyond 28 days; however, found no significant impact due to age with sand replacement for waste glass. Samples of different replacements followed a similar trend as that of the control sample containing natural aggregate (NA), illustrated in Fig. 5.

Past researchers have found variable results on the mechanical properties due to the incorporation of waste glass. Positive impacts on the compressive strength can be attributed to fine glass particles acting as filler material providing additional hydration sites for the cement or the pozzolanic activity which is exhibited by glass. Fine glass particles are able to provide additional hydration sites for the cement particles, increasing the cement hydrates developed while exhibiting the pozzolanic activity due to the silica within the glass. The pozzolanic activity develops additional C–S–H gel to that produced from the cement powder. This combined effect has the potential to increase the compressive strength of mortars or concretes; however, negative impacts can arise due to the dilution effect when a larger quantity of cement is substituted for glass, where insufficient amounts of calcium carbonate are available to react with the silica within the glass to produce more C–S–H gel. The effective mechanical properties of cement-based materials incorporating waste glass come from a combination of these effects, where it is found that lower replacements of finer glass particles may increase the compressive strength, however, increasing the particle size of the glass or replacement level may yield unsatisfactory results. Other potential sources for the variability in results may include the grade of glass used, type of glass, or type of natural aggregate used. A summary of the effects on the mechanical properties can be found in Table 4. Mortars obtaining sufficient strength values can be used in masonry to bind bricks together; however, lower strength mortars containing sufficient amounts of glass have the possibility to be used as exterior cladding for the aesthetic appeal due to the glass.

**Table 4:** Summary of the effects of waste glass on the mechanical properties of concretes and mortars.

Replacement [%]	Particle Size [μm]	Compressive Strength				Flexural Strength				Tensile Strength	Ref.	Notes
		3	7	28	>28	3	7	28	>28	28		
20, 50	83% < 1190	–	–	↑	↑	–	–	↑	↑	–	[31]	
100				↓	↑			↑	↑			
20	3000	–	↑	↑	↑	–	–	↑	–	↑	[32]	56-days
40			↓	↓	↓			↓		↑		
60			↓	↓	↓			↑		↓		
30	36	–	–	–	↑	–	–	–	∼	–	[33]	180-days
	36-50				↑				∼			
	50-100				↑				↑			
70	36,36–50,50-100				↑				↑			
25, 50, 75, 100	<600	–	–	↑	–	–	–	–	–	–	[34]	
	>600			↓								
5, 10, 15, 20	90% < 4750	–	–	↑	–	–	–	↑	–	–	[35]	
40, 100	81% < 4750	–	–	–	–	–	–	–	–	–	[36]	
10, 20, 30, 40	75-100	–	–	↑	–	–	–	–	–	–	[37]	
20	150	∼	∼	∼	–	–	–	–	–	–	[27]	
10, 30, 40, 50		↓	↓	↓								
30, 50, 70	600	–	–	↓	–	–	↓	↓	↓	↓	[38]	91-days
50, 100	87% < 2360	–	–	↓	–	–	–	↓	–	–	[39]	
10, 20, 30, 40, 50	64% < 1180	–	↓	↓	–	–	–	↓	–	↓	[40]	
15, 30, 45, 60	85% < 2000	–	–	↓	–	–	–	–	–	↓	[41]	
25	95% < 1600	↓∗	↓	↑	–	↑∗	↑	↓	–		[42]	∗2 -days
50, 75, 100		↓∗	↓	↓		↑∗	↓	↓				
25	90% < 2360	–	↓	↓	–	–	↓	–	–		[43]	
50, 75, 100			↓	↓			↓	↓				
10, 20, 30, 40, 50	60% < 1000	–	↓	↓	–	–	↓	↓	–		[44]	
20	17	–	–	↓	–	–	–	↑	–		[45]	
5, 10, 15	600	–	∼	∼	∼	–	–	∼	–	–	[46]	
20, 30, 50			↓	↓	↓			↓				
10	89% < 2360	↑	↑	↓	–	↑	↑	↑	–	–	[26]	
15, 20		↑	↑	↑		↑	↑	↑				
50	150 - 4750	–	↑	↑	–	–	–	–	–	–	[28]	
10, 20, 30, 40	92.4% < 15		–	↑								

‘↑’ denotes sample improved relative to reference sample; ‘↓’ denotes sample decreased; ‘∼‘denotes negligible results to reference.

#### 4.1.2 Effects of fine aggregate replacement on fresh properties

The workability of a concrete mix can be determined by various tests of fresh samples, including a slump test, compaction factor test, or a vee-bee consistency test. The results from the literate show that increasing the waste glass replacement for natural sand has a decreasing effect on the slump [26,37,[44], [45], [46]]. Although glass is smooth, this is attributed to the lighter weight of the samples as glass has a lower specific gravity than that of sand. Mardani-Aghabaglou et al. [41] and Ali and Al-Tersawy [40] found an increasing slump with an increasing replacement ratio. The authors attribute this to the glass particles being finer than that of sand, allowing for a higher compaction. Due to the lower specific gravity of glass, the authors also found that samples with increasing replacements of waste glass had lower fresh and dry densities. The effects of waste glass on the fresh properties of concretes and mortars are summarised in Table 5.

**Table 5:** Summary of the effects of waste glass on the fresh properties of concretes and mortars.

Replacement [%]	Particle Size [μm]	Slump	V-Funnel	Density	Ref.
20,50,100	83% < 1190	–	–	↓	[31]
15,30,45,60	80% < 2000	↑	–	↓	[41]
20	17	↓	–	↓	[45]
5,10,15	600	↓	–		[46]
10,20,30,40,50	60% < 1000	↑↓↑	↑	↓	[44]
10,20,30,40,50	64% < 1180	↑	↑		[40]
10,20,30,40	75–100	↓	–	–	[37]
10,15,20	89% < 2360	↓	–	↓	[26]

### 4.2 Coarse aggregate replacement

Waste glass has the potential to be utilized as coarse aggregate in concretes which may bring aesthetical benefits; however, with a potential loss to the mechanical properties. Afshinnia and Rangaraju [45] found replacing natural coarse aggregate for coarse waste glass considerably reduced the compressive and splitting tensile strengths, with a reduction of 38% for the compressive strength. This is supported by the findings produced by Topcu and Canbaz [47], and Kou and Poon [48] who found that replacing traditional coarse aggregate for waste glass can have detrimental effects on the compressive and splitting tensile strengths while having negligible effects on the workability. Due to the smooth surface area of the glass, a weaker bond forms between the crushed glass aggregate and the cement paste, effecting the developed strength. Gerges et al. [36] found increasing the replacement ratio of coarse waste glass decreases the 7-day and 28-day compressive strengths. Samples incorporating coarse waste glass has a larger difference in compressive strength at 7-days than at 28-days compared to the control. This indicates late strength development which is produced in the waste glass samples. Terro [49] found that replacements of coarse aggregate up to 10% (by volume) can produce higher compressive strengths at temperatures greater than 150 °C, however, increasing the replacement over 10% causes a decrease in compressive strength with extreme temperatures. The author also found that waste glass as a fine aggregate produce more promising results than when waste glass is used as coarse aggregate. Topcu and Canbaz [47] found that an increase in waste glass aggregate increased the Ve-Be consistency test values. Giving a decrease in slump, however, an increase in consistency shows that the incorporation of waste glass has negligible effects on the workability. In this regard, it was found an increase in slump which was attributed to the smoothness of the glass surface, provides poor interference with the mix [49]. A summary of the effects due to waste glass as coarse aggregate can be seen in Table 6.

**Table 6:** Summary of effects due to waste glass as coarse aggregate.

Replacement [%]	Particle Size [μm]	Compressive Strength	Splitting Strength	Workability	Ref.
80,100	4750–9500	↓↓	↓		[45]
15,30,45,60	4000–16000	↓	↓	↓	[47]
15,30,45	5000–14000	↓	↓	–	[48]
33,50,67,100	60% < 7620	↓		↓	[36]
10,25,50,100	9525–19,050	↓		–	[49]

## 5 Future research directions and conclusion

This review aimed to investigate the effects which incorporating waste glass into cement-based materials may have on the fresh and hardened properties of mortars and concretes. More research needs to transpire to investigate the optimal dosage of waste glass as a fine aggregate replacement, considering the particle size and replacement percentages. The past research primarily focused on the effects of one type of class, being clear container glass; however, due to cross colour contamination, mixed colours cannot be recycled using traditional methods. Therefore, more research should be completed to investigate the effects which a mixture of clear, amber, and green container glass may have on the fresh and hardened properties of concretes or mortars. Glass is a common material that can be recycled indefinitely to produce new glass products. However, a large quantity of glass waste is sent to landfill either due to improper recycling procedures by the consumer or overloading of the recycling plants. The construction industry is one of the largest polluters in the world due to the production of cement and cement-based materials; strong changes are needed to create a more sustainable future for cement-based materials. This literature review analysed past research into the effects of incorporating waste glass in the cement process. Silica is the main ingredient of glass, which may support the alkali-silica reaction. However, if the glass is milled to a fine powder, a positive pozzolanic reaction takes place which strengthens the concrete while depleting the alkali. The literature shows that waste glass produces unfavourable results when used as a raw material replacement in the production of clinker but as a partial clinker replacement, it has a positive impact on the mechanical properties due to the pozzolanic reaction that occurs. However, the large replacements of waste glass for cement can hinder the compressive strength due to the dilution effect, where inadequate levels of calcium carbonate remain to produce the C–S–H gel. The research studies show that 20% of cement can be replaced by waste glass of 20 μm without hindering the mechanical properties. However, waste glass as a fine aggregate has an increasingly adverse effect on the mechanical properties as the replacement of sand increases over 20%. In terms of fresh properties, due to the lower specific gravity of glass compared to natural sand, samples made incorporating waste glass has a lower slump density. Waste glass is a poor coarse aggregate replacement due to glasses smooth surface area. This hinders the bond strength developed between the cement paste and the glass aggregate. To allow waste glass in cement-based materials to become a commercially viable product, more research needs to be undertaken in the mix design to minimise the likelihood of the alkali-silica reaction from occurring while optimizing the pozzolanic activity produced from the glass. With the right mix design, waste glass has the potential to be utilized in all forms of construction. From the past research it is seen that low replacements of cement or fine aggregate for waste glass containing finer particles can be beneficial; however, there are still contradictory results that show the optimal mix design has still not been found.

## Declaration of competing interest

The authors declare that they have no known competing financial interests or personal relationships that could have appeared to influence the work reported in this paper.
